# BOOTStrap-SCI: Beyond One option of treatment for spinal trauma and spinal cord injury: Consensus-based stratified protocols for pre-hospital care and emergency room (part I)

**DOI:** 10.1016/j.bas.2025.104251

**Published:** 2025-04-04

**Authors:** Nicolò Marchesini, Andreas K. Demetriades, Oscar Alves, Riya Mandar Dange, Harold Mauricio Choco, Edinson Dussan Lozada, Dumar Javier Figueredo Sanabria, Angélica Gamboa, Luz Llined Mendoza Victoria, Enoc Noscue Montealegre, Jonathan A. Pardo Carranza, Jonathan Velásquez Quintero, Andrès M. Rubiano, the BOOTStraP-SCI Study Group

**Affiliations:** a Department of Neurosurgery, Azienda Ospedaliera Universitaria Integrata di Verona, Verona, Italy; b EANS Global and Humanitarian Neurosurgery Committee, International; c Department of Neurosurgery, Royal Infirmary Edinburgh, NHS Lothian, Edinburgh, United Kingdom; d Department of Neurosurgery, Hospital Lusíadas Porto, Porto, Portugal; e University of Pittsburgh School of Medicine, Pittsburgh, PA, United States; f Universidad del Valle, Cali, Colombia; g Sistema Nacional de Bomberos de Colombia, Bogotà, Colombia; h Emergency Service Hospital Simon Bolivar, Bogotà, Colombia; i Fundación Meditech, Cali, Colombia; j Centro de Servicios de Salud Regional, Antioquia, Colombia; k Secretaria de Salud, Neiva, Colombia; l Universidad Militar Nueva Granada, Bogotà, Colombia; m Servicio de Medicina de Urgencias y Emergencias, Fundación Valle del Lili, Cali, Colombia; n Universidad El Bosque, Bogotá, Colombia

**Keywords:** Spinal trauma, spinal cord injury, Protocols, Low- and middle-income countries (LMICs), Pre-hospital care, Emergency room

## Abstract

**Introduction:**

Spinal trauma (STx), with or without spinal cord injury (SCI), represents a significant global health burden, particularly in low- and middle-income countries (LMICs). Existing guidelines often rely on tools and resources that are not always universally available, especially in less resourced settings, contributing to disparities in care and outcomes. A pragmatic, resource-adapted approach may help optimize management in these contexts.

**Research question:**

This study aimed to develop resource-adapted protocols for pre-hospital and emergency room management of STx and SCI, addressing challenges specific to LMICs while supported by clinical evidence and expert based practices.

**Material and methods:**

A multidisciplinary Delphi consensus combined international evidence-based guidelines with expert opinions. Iterative discussions and voting by healthcare providers from LMICs and high-income countries (HICs) ensured the development of context-sensitive protocols. These were tailored to varying levels of training, resource availability, and healthcare infrastructure.

**Results:**

The resulting protocols address key areas of pre-hospital and emergency management, including initial resuscitation, immobilization, clinical interventions, and timely referral. These protocols emphasize adaptability, providing structured plus flexible guidance for optimizing care according to specific contexts from low to high resourced clinical settings.

**Discussion and conclusion:**

The proposed protocols are not intended as gold-standard guidelines but as adaptable frameworks to guide management of STx/SCI in contexts with different availability of resources. By addressing disparities in resource availability and clinical competencies, they can serve as a foundation for local adaptations and improvements in care. Future research should evaluate their implementation and impact on outcomes.

## Highlights

• Context-specific protocols for spinal trauma and SCI management in LMICs were developed.
• Step-by-step guidelines address hemodynamic management, imaging, and surgical decision-making.
• Protocols are stratified for basic, intermediate, and advanced resource settings.
• A consensus-driven approach ensures feasibility and adaptability in LMICs.
• Future validation of these protocols in clinical practice is essential.

## Abbreviations:

**LMICs**: low-and middle income countries
**HICs**: high-income countries
**SCI**: spinal cord injury
**ATLS**: advanced trauma life support
**AVPU**: Alert, Verbal, Painful, Unresponsiveness
**BVM**: bag-valve-mask
**TBI**: traumatic brain injury
**GCS**: Glasgow Coma Scale

## 1 Introduction

Spinal trauma (STx), associated or not with spinal cord injury (SCI), represents a significant global health challenge, with disproportionately higher burden in low- and middle-income countries (LMICs) (Kumar et al., 2018). Indeed, it is estimated that the number of persons affected by STx per annum is more than 800.000 less resourced regions, while approximately 100.000 in high-income countries (HICs) (Kumar et al., 2018). Beyond medical issues, STx and SCI carry immense direct and indirect social and economic consequences, for individuals, caregivers and healthcare systems (Cao and Krause, 2020; Soendergaard et al., 2022).

While the burden of STx in LMICs is enormous due to higher incidence rates, this challenge is further exacerbated by systemic barriers which often hinder timely and adequate care. These barriers include limited access to specialized services, insufficient training, delays in reaching healthcare facilities and consequently diagnostic and treatment delays, inadequate diagnostic and therapeutic resources (Mukhopadhyay et al., 2019; WHO. World Health Statistics, 2021; Azad et al., 2023).

These issues contribute to significant disparities in treatment quality and outcomes between regions (Chiu et al., 2010). Specifically, in the pre-hospital and emergency room phases of care, factors such as the availability of trained personnel, timely transportation, imaging modalities, and basic life support can vary greatly, further exacerbating disparities (Azad et al., 2023; Yohann et al., 2022).

Present international guidelines represent a synthesis of the best available evidence. They are designed to optimize care pathways and improve outcomes, potentially helping to bridge disparities among various healthcare contexts. However, they are typically developed in high-resource settings and often don't account for the diverse realities of low-resourced environments. In other words, these guidelines frequently assume the direct or indirect availability of infrastructures, tools, techniques, personnel, and resources that in the real world where most STx occurs may be inadequate or entirely absent (Maschmann et al., 2019; Yue et al., 2017; Sharif et al., 2020; Sánchez et al., 2020; Alves et al., 2020; Peev et al., 2020; Zileli et al., 2020a, 2020b).

A pragmatic approach involving the development of protocols of care tailored to the resources available in specific contexts has been previously proposed for traumatic brain injury and other topics (Rubiano et al., 2020; Wilson et al., 2022; Duggan et al., 2020). This strategy in neurotrauma (proposed by our group and currently on impact evaluation for the BOOTStraP-TBI) is based on optimizing care within existing limitations. In a prior study exploring the interest towards this approach, over 95 % of spinal surgeons in LMICs were very supportive of it (Marchesini et al., 2022a).

Based on this background, we aimed to create protocols for the management of STx and SCI that may be adaptable to different levels of resources. Using a consensus-driven methodology grounded in available evidence, we sought to capture diverse perspectives and develop practical recommendations that address the unique challenges of different settings, taking advantage of the expertise of people that already work in high-volume centers in different settings with and without limitations of resources of care.

In this article we present the methodology we employed and discuss the final protocols on pre-hospital and emergency room care. The protocols for the intensive care unit (ICU) and surgical management will be discussed in a parallel article included in this issue.

## 2 Methods

This study followed a three-phase methodology adapted from a prior initiative involving some of the authors: consensus preparation, a live consensus meeting, and a post-consensus phase (Rubiano et al., 2020). A steering committee of four researchers (NM, AMR, AD, OA) was responsible for designing and supervising the process. The methodology was formally presented to the participants by the methodological group of the Meditech Foundation in Colombia, during the XVII World Congress of Neurosurgery (WFNS, 2022 Bogotà) and endorsed by the WFNS (World Federation of Neurosurgical Societies) Neurotrauma Committee and supported by the EANS (European Association of Neurosurgical Societies) 10.13039/100008086Global and Humanitarian Neurosurgery Committee.

### 2.1 Consensus preparation

A multidisciplinary panel of experts with competencies on each of the four phases of STx care—pre-hospital, emergency room, intensive care, and surgery—was assembled. Panelists were purposely selected by the steering committee to ensure diverse expertise and prioritize representation from LMICs, particularly those with experience managing STx in resource-constrained settings. Selection criteria included demonstrated expertise in STx management through clinical roles and/or prior contribution to guideline development. The steering committee also ensured diversity and balance in clinical versus academic representation to capture a broad range of perspectives.

The steering committee developed a set of open-ended questions to guide the discussions during the live consensus. These questions were designed to encourage panelists to propose context-specific management strategies tailored to different scenarios and, within each scenario, resource availability. Specifically, the questions asked to develop step-by-step protocols of management, in a hierarchical process. To define the level of resources for each scenario, the same classification used in the previous study on traumatic brain injury (TBI) for prehospital care and emergency room settings was adopted (Rubiano et al., 2020).(Table 1) For the ICU (Intensive Care Unit) and surgical phases (presented in a different article), a greater emphasis was placed on imaging capabilities, as the management of STx often heavily depends on the availability and sophistication of imaging modalities.

**Table 1:** General scenarios defined for the development of stratified protocols in the management of STx with or without SCI.

What is the best step-by-step protocol for treating an adult patient with suspected spinal trauma in a:	
**Prehospital care**	1. Basic emergency transport 2. Advanced emergency transport
**Emergency Room**	1. Low-complexity facility (without CT scan) 2. Medium-advanced facility (with CT scan)
**Intensive Care Unit**	1. Post-operative recovery area 2. Intermediate Care Unit-General Intensive Care Unit
**Surgery**	1. Low-complexity facility (without imaging) 2. Medium-complexity facility (with only x-ray) 3. Medium-high complexity facility (with CT w/o MRI)

A literature search of guidelines, protocols, and recommendations related to STx and SCI across the four care phases was conducted. The search, limited to articles published in English from 2011 to December 2021, used the following keywords and combinations: *guideline*, *protocol*, *recommendations*, *spinal trauma*, *spinal cord injury*, *pre-hospital care*, *emergency care*, *emergency department*, *treatment*, *surgery*, *neurosurgery*, and *intensive care*. Articles were screened for relevance by the steering committee members with experience in neurotrauma care and guideline development (AMR, AD, OA).

Selected articles were organized into four thematic folders corresponding to each care phase. One month before the live consensus meeting, all participants were provided with access to these materials along with written instructions detailing the process. Participants were encouraged to review the materials relevant to their field, as these documents constituted the scientific foundation of future discussions.

### 2.2 Consensus meeting

The in-person consensus meeting took place on March 13, 2022, in Bogotá, Colombia, using a modified Delphi approach.

**Phase 1**: Panelists were grouped by field of expertise (e.g., pre-hospital, emergency care) and worked collaboratively at separate tables, each facilitated by a table moderator. Before the meeting, all moderators received information on facilitation techniques, emphasizing impartiality, encouraging balanced participation, and managing time effectively. This training ensured consistency across all group discussions. Discussions were recorded, and detailed notes were taken by an assigned staff member. Panelists shared personal experiences, perspectives, and evidence to draft step-by-step protocolized recommendations for their specific care phase based on the guiding questions. Each proposed protocol step required a minimum of 70 % agreement within the group to advance.

**Phase 2**: The groups convened in a joint plenary session. A representative from each group presented their drafted protocols to the entire panel. During this session, all participants were encouraged to provide feedback to refine the protocols. Final approval of each protocol required 100 % consensus among all panelists. If full consensus was not initially achieved, iterative re-voting and re-discussion continued until unanimity was reached. To ensure transparency, individual voting records were anonymized, and final protocols were documented with a summary of key revisions made during each voting round.

### 2.3 Post-consensus phase

The outputs of the consensus meeting were reviewed and refined by the steering committee. Draft protocols were formatted into modifiable PDF documents, with each step accompanied by "agree/disagree" options and space for open comments. These documents were circulated to all panelists for final validation, focusing on identifying and rectifying substantial errors. Disagreements related solely to language, phrasing, or procedural order were not considered valid grounds for revision unless accompanied by explanatory comments.

After collecting the responses, the final outputs consisted of the BOOTStraP-SCI protocols. The step-by-step protocols were converted into algorithms to facilitate easier comprehension and visualization.

### 2.4 How to interpret and use the proposed protocols

**Fig. 1:**
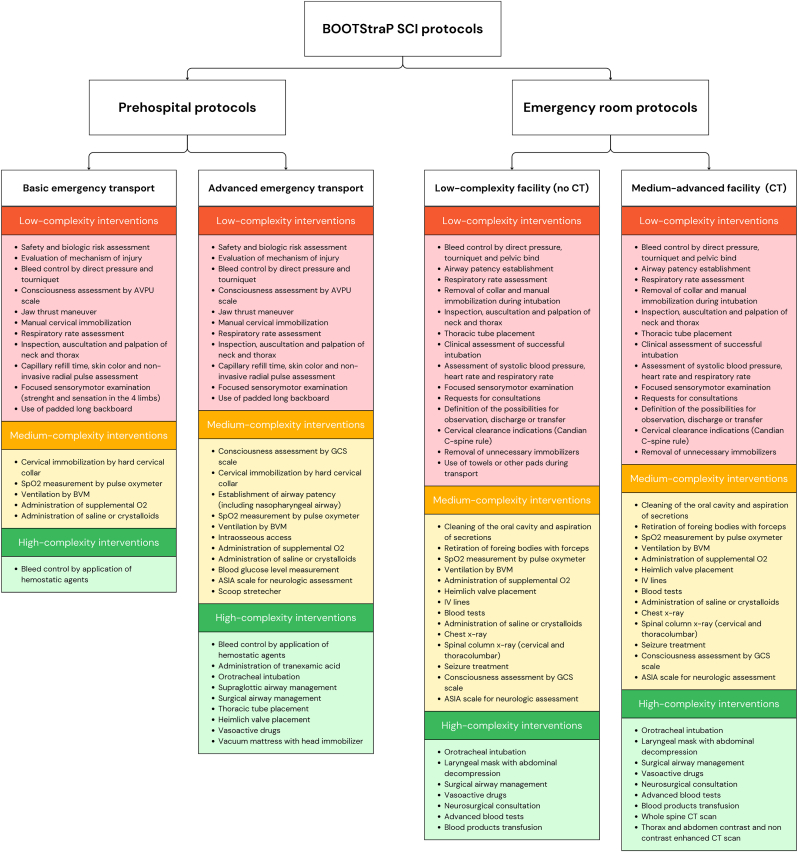
General scheme of the stratified interventions for spinal trauma management protocols for low- and high-resource facilities, illustrating the prehospital and emergency room (ER) care pathways. Each protocol is subdivided into two scenarios: basic and advanced emergency transport for prehospital care, and low-complexity and medium-advanced facilities for ER care. Within each protocol, interventions are categorized by complexity—low (red), medium (yellow), and high (green)—to guide healthcare providers in selecting appropriate actions based on the resources, infrastructure, and expertise available in their facility.

For each treatment phase, we developed hypothetical scenarios (our questions) to reflect real-world situations, acknowledging that specific details may vary across different contexts. As such, the proposed protocols are intended as flexible frameworks rather than rigid classifications, stratified by the available resources and possibilities for interventions. Within each treatment phase, scenarios are broadly categorized into specific contexts and then protocols are defined. Then, each protocol is stratified by the resources available, enabling customization of interventions to suit every context (Fig. 1). This approach allows healthcare providers in any of the four treatment phases to a) Identify the protocol that aligns with their facility's capabilities, and b) select the most appropriate intervention within that protocol. To enhance clarity, treatment options are visually represented using a color-coded system: red indicates interventions that can be performed with minimal resources; yellow indicates interventions suitable for settings with moderate resource availability and green represents interventions typically performed in facilities with advanced resources. All proposed interventions can be selected from the algorithms and are organized across different levels of care based on the availability of the outlined resources.

## 3 Results

In this article, the pre-hospital and emergency room algorithms for the protocols are presented, to allow better comprehension of the early phases of care. The complete version of the protocols (including the step-by-step recommendations) is detailed as supplementary material. The Intensive Care and Surgery protocols will be published elsewhere.

**Fig. 2:**
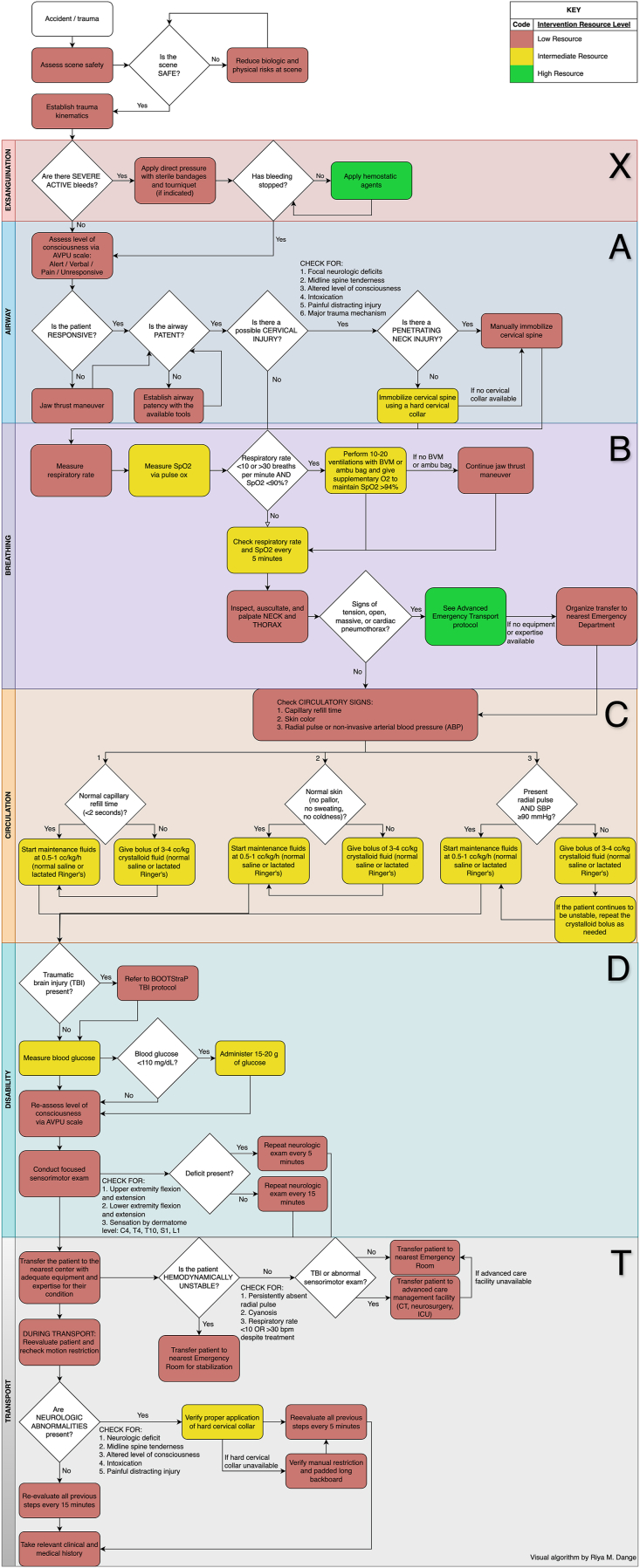
Algorithm for the management of an adult with suspected spinal trauma in a Basic Emergency Transport.

**Fig. 3:**
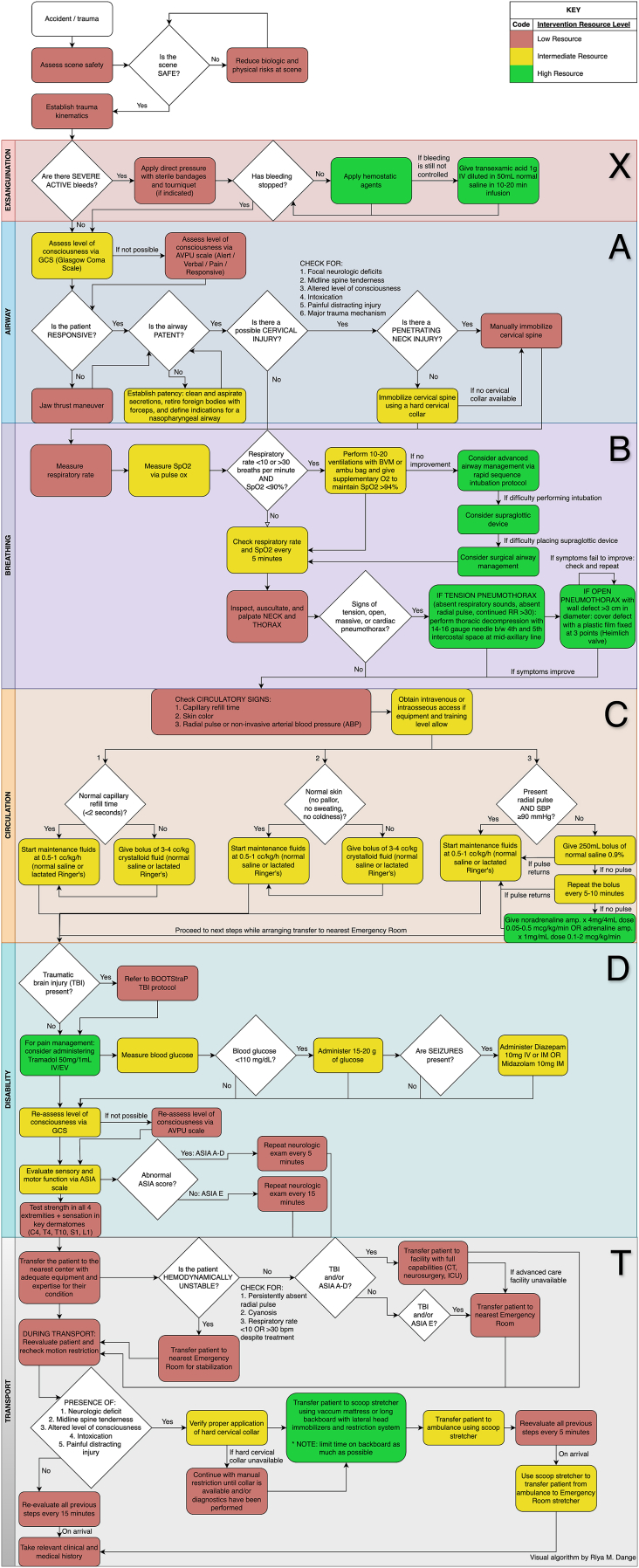
Algorithm for the management of an adult with suspected spinal trauma in an Advanced Emergency Transport.

**Fig. 4:**
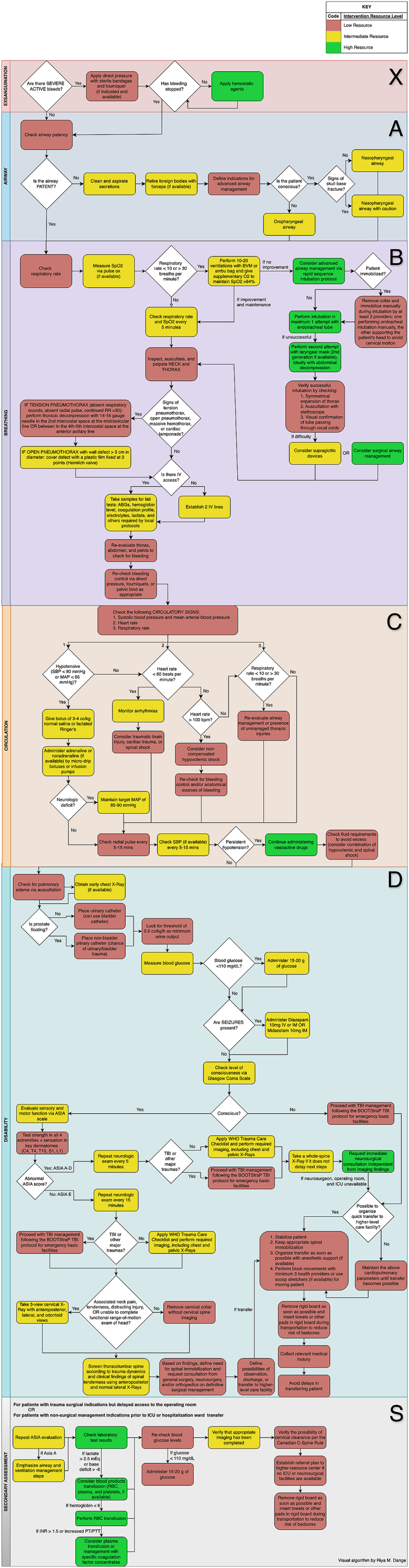
Algorithm for the management of an adult with suspected spinal trauma in a Low-complexity facility (without CT scan).

**Fig. 5:**
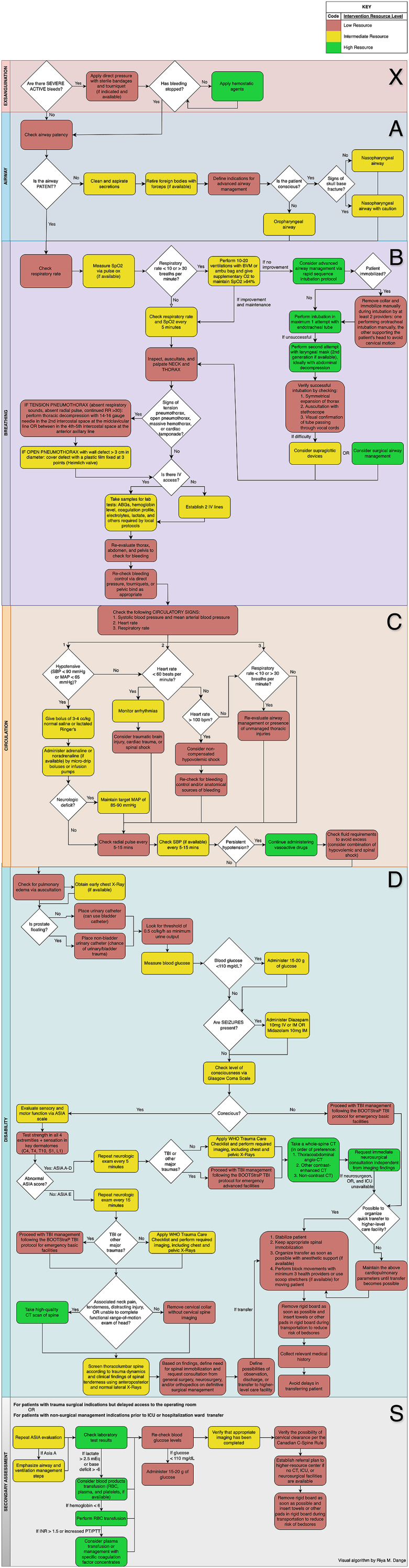
Algorithm for the management of an adult with suspected spinal trauma in a medium-advanced facility (with CT scan).

In this Results section, only a narrative version of the protocols is reported. For a comprehensive understanding, the reader should refer to the original protocols (Supplement I and Supplement II) and the associated algorithms (Fig. 2, Fig. 3, Fig. 4, Fig. 5).

### 3.1 Pre-hospital protocols and associated algorithms

The pre-hospital protocols were divided into two scenarios: a) Basic Emergency Transport and b) Advanced Emergency Transport.

a) *What is the best step-by-step protocol for treating an adult patient with suspected spinal trauma in a Basic Emergency Transport* (Fig. 2)?

The protocol begins with **scene safety verification**, followed by the resuscitation process guided by the ATLS x-ABC principles suggested by the standard trauma approaches. The importance of prioritizing circulation management, as emphasized in several standardized trauma care training programs like the Basic Emergency Course (10.13039/100004423World Health Organization) or the Pre-hospital Trauma Life Support (10.13039/100025245National Association of Emergency Medical Technicians and the Committee on trauma of the 10.13039/100005301American College of Surgeons), reflects growing evidence supporting its role in reducing trauma mortality (Ritondale et al., 2024).

I. **Exsanguination**: Severe bleeding should be controlled using direct pressure before proceeding to subsequent steps. Providers are advised not to delay at this stage if life-threatening hemorrhages persist.
II. **Level of Consciousness**: Consciousness should be assessed using the Alert/Verbal/Pain/Unconsciousness (AVPU) scale.
III. **Airway Management**: For patients with suspected STx, the jaw-thrust maneuver is recommended over the head-tilt/chin-lift technique to maintain airway patency while minimizing spinal movement. Airway tools should be used based on local availability.
IV. **Spinal Precautions**: Indicators for potential spinal injury include focal neurological deficits, midline spine tenderness, altered consciousness, intoxication, distracting injuries, or a high-energy trauma mechanism. In such cases, spinal precautions must be maintained throughout care. Manual immobilization is advised for penetrating neck injuries, while hard cervical collars should be used for other suspected spinal injuries (if available).
V. **Breathing Management**: Providers should assess respiratory rate and, if available, oxygen saturation. Management options include bag-valve-mask ventilation and supplemental oxygen (if available). Inspection, palpation, and auscultation of the neck and thorax should identify severe injuries, with stabilization and referral plans established accordingly.
VI. **Circulation Assessment**: Capillary refill time, skin color, and radial pulse are recommended diagnostic measures, with fluid management initiated as needed.
VII. **Neurological Assessment**: A focused evaluation for spinal cord injury includes strength testing of all four limbs and sensory assessment by dermatome. Abnormal findings warrant immediate referral to a facility equipped for SCI management.
VIII. **Transport and Reassessment**: During transport, periodic re-evaluation of the patient's condition and proper immobilization must be maintained according to the clinical findings.

For cases with concurrent traumatic brain injury (TBI), providers are referred to previously established protocols for TBI management (Rubiano et al., 2020).

b) *What is the best step-by-step protocol for treating an adult patient with suspected spinal trauma in a Basic Emergency Transport* (Fig. 3)?

The protocol includes the main same recommendation from the basic emergency transport protocols but adds some steps as per higher resources available.

I. **Exsanguination:** Administration of tranexamic acid is recommended for uncontrolled severe bleeding (if available).
II. **Level of Consciousness**: Consciousness should be assessed using the Glasgow Coma Scale (GCS).
III. **Airway Management:** While similar to the Basic protocol, advanced airway management using endotracheal tubes or supraglottic devices is indicated if BVM ventilation and supplemental oxygen are insufficient.
IV. **Spinal Precautions:** Recommendations remain the same as the Basic Emergency Transport protocol.
V. **Breathing Management**: Additional guidance is provided for advanced airway interventions in cases of respiratory compromise. Emergent interventions are specified for severe neck or thoracic injuries.
VI. **Circulation Assessment**: Hemodynamic instability warrants the use of vasopressors based on specific clinical criteria.
VII. **Neurological Assessment**: the American Spinal Injury Association (ASIA) scoring system is recommended for comprehensive neurological evaluation.
VIII. **Transport and Reassessment**: Additional immobilization tools and advanced transport equipment are introduced for enhanced patient stabilization during transfer.

### 3.2 Emergency room protocols

The emergency room protocols were divided into two scenarios: a) low complexity facility and b) medium-advanced facility.

a) *What is the best step-by-step protocol for treating an adult patient with suspected spinal trauma in a low-complexity facility? (without CT scan)* (Fig. 4)
  I. **Exsanguination:** Severe bleeding should be controlled using direct pressure before proceeding to subsequent steps. Providers are advised not to delay at this stage if life-threatening hemorrhages persist.
  II. **Airway Management**: Recommendations include using oropharyngeal or nasopharyngeal airways based on the patient's level of consciousness. Specific indications are provided for invasive airway management (if available) and temporary clearance of cervical collars during airway management.
  III. **Breathing Management**: Respiratory rate and oxygen saturation (if available) are assessed. Management options include bag-valve-mask ventilation and supplemental oxygen. Severe thoracic or neck injuries are managed through stabilization and planning for referral.
  IV. **Circulation Assessment**: Systolic blood pressure, heart rate, and respiratory rate are used for circulation assessment. Fluid resuscitation and the initiation of vasoactive drugs are indicated for hemodynamic instability.
  V. **Disability**: Neurological evaluation involves strength testing of all four limbs and sensory assessment by dermatome. Patients with abnormalities require immediate imaging and neurosurgical consultation (if available). For patients without neurological deficits, criteria for further imaging are detailed.
  VI. **Spinal Precautions clearance**: Clinical and radiographic parameters are outlined to determine clearance from immobilization devices.
  VII. **Transport and Reassessment**: Periodic re-evaluation of the patient's condition is required during transfer, ensuring proper immobilization throughout.
b) *What is the best step-by-step protocol for treating an adult patient with suspected spinal trauma in a medium-advanced facility (with CT scan)* (Fig. 5)

The medium-advanced facility protocol builds on the low-complexity protocol (defined previously) with these additional adjustments according to the availability of CT scanning:

**Imaging**: Indications for obtaining a CT scan are specified, including the presence of focal neurological deficits, severe trauma mechanisms, or inconclusive clinical evaluations.

**Management Alignment**: No significant changes were introduced for the remaining protocol steps, which align with those in low-complexity facilities. However, CT imaging facilitates more accurate diagnosis and decision-making regarding spinal precautions and further interventions.

## 4 Discussion

In this article, we present a set of resource-adapted protocols for the prehospital and emergency room management of STx and SCI. Developed through a multidisciplinary consensus-building process grounded in available evidence, these protocols provide flexible and pragmatic frameworks to optimize patient care tailored to available resources.

Studies have shown that outcomes for traumatic spinal injuries differ significantly between HICs and LMICs. In HICs, first-year survival rates range from 94.0 % to 95.6 %, with in-hospital mortality between 2.1 % and 7.0 %. In contrast, studies from LMICs report nearly three times higher in-hospital mortality, reaching up to 24.1 % in Africa, and first-year survival rates as low as 86.5 % in some regions of the Americas (Eisner et al., 2021). Several key factors contribute to these disparities, including access to specialized spinal injury centers, delays in surgery, the level of equipment in facilities, and the availability of comprehensive prehospital and rehabilitation services (Jin et al., 2021). These data highlight a clear association between healthcare system factors and the observed differences in outcomes for traumatic spinal injuries.

Specifically for pre-hospital care, a significant proportion of patients with STx lack access to emergency services globally. The fragmentation and inadequacy of pre-hospital systems, particularly in LMICs, can have profound effects on outcomes. For this reason, improving prehospital care is widely recognized as crucial for reducing mortality and disability (Eisner et al., 2021; Jin et al., 2021). Effective resuscitation and the maintenance of cardiopulmonary parameters are critical, especially in SCI cases where adequate oxygenation and spinal cord perfusion pressure must be ensured (Oteir et al., 2014). Accordingly, current guidelines provide explicit values and timeframes for these measures (Sánchez et al., 2020; Ryken et al., 2013). However, adherence to these recommendations can be limited by a lack of trained personnel or resources in many regions (Marchesini et al., 2022a). Indeed, prehospital care systems in LMICs may be disorganized and lack cohesion, with deficiencies in trained medical personnel and first responders, insufficient essential resources, and inadequate infrastructure, particularly when contrasted with those in HICs (McDonald et al., 2016).

The protocolized steps proposed in our protocols are adaptable to providers with varying levels of training, enabling them to recognize critical situations and implement appropriate interventions, even in resource-limited settings.

The literature on prehospital immobilization and transport of patients with suspected STx/SCI is sparse and often controversial (McDonald et al., 2016; Ahn et al., 2011). Most guidelines still include different approaches to ensure spinal alignment and spinal cord protection during extrication, mobilization and transfer of these patients (Maschmann et al., 2019; Zileli et al., 2020b; Hawkins et al., 2019; Kornhall et al., 2017; Fischer et al., 2018; Velopulos et al., 2018; Theodore et al., 2013). However, regular use of cervical collars or spinal backboards is reported by less than half of spinal healthcare providers in LMICs overall, with significant inter-region differences correlating to income area. For example, the routine use of a hard cervical collar was reported by only 6 % of providers in low-income countries and 26 % in lower-middle-income countries (Demetriades et al., 2022). In contrast, this figure exceeds 80 % in HICs, highlighting a stark disparity in prehospital spinal injury management (Muzyka et al., 2024). Contributing factors include inadequate training and limited access to equipment such as cervical collars and spinal backboards. By offering tiered immobilization strategies within our protocols, we aim to enhance training and improve adherence, even when not all equipment is readily available.

Early clinical identification and classification of patients with SCI, including milder cases, is critical for prognostication and to guide timely patient transfers to the most appropriate definitive center of care (Azad et al., 2023). For these reasons, guidelines recommend the systematic adoption of the American Spinal Injury Association (ASIA) classification standard for assessing neurological and functional impairment (Sánchez et al., 2020; Hadley et al., 2013). However, competencies in neurological assessment can vary significantly across contexts, and simpler tools may be the only feasible option in certain regions. The introduction within the protocols of steps for neurological assessment that consider different competencies may be helpful for the identification of patients that require specialized care and quick transfer. Moreover, these protocols could serve as foundational tools for formal training programs, which have been shown to enhance the accuracy of neurological classification (Chafetz et al., 2008).

Most of the literature underscores the importance of a timely definitive treatment in patients with STx and neurological impairment (Badhiwala et al., 2021). Delays, particularly common in LMICs, have been associated with poorer outcomes also in neurologically intact patients (Carreon and Dimar, 2011). A systematic review found that nearly 50 % of patients in LMICs presented more than 24 h post-injury, compared to only 12 % in HICs (Azad et al., 2023). In certain regions, delays are measured in days, rather than hours (Magogo et al., 2021). Several aspects of pre-hospital care can affect the timing of treatment, with prolonged transfer times being reported as the most frequent issue (Marchesini et al., 2022b). Indeed, a previous study highlighted substantial variations in the time to presentation for care across different income and geographic regions, with nearly two-thirds of healthcare providers in low-income areas reporting that they receive SCI patients more than 24 h after the initial trauma. This contrasts with most HICs, where the majority of cases are referred to a definitive care center in a timely manner (Demetriades et al., 2022). One possible factor contributing to delays in presentation is the limited availability of rapid transportation, such as air transport. A recent survey estimated that only 8 % of providers in low-income regions have access to air transportation for SCI patients, while the availability remains low even in upper-middle-income countries, at just 40 % (Demetriades et al., 2022). The choice of transportation should be adapted to the specific needs of the intervention setting to optimize survival outcomes and promote optimal recovery (Lapidus et al., 2023). All that being said, evidence on the impact of time on outcomes supports the recommendation that patients with traumatic SCI should be transported to a definitive care center as quickly as possible (Yohann et al., 2022; Sánchez et al., 2020). While some delays are unavoidable, structured protocols, such as ours, may help prioritize urgent transfers and facilitate decisions about the most appropriate destination for definitive care. The inclusion of scenario-specific transport recommendations in our protocols aims to address these challenges and improve timeliness to treatment.

In the emergency room, interventions for trauma patients with suspected spinal injuries span resuscitation, immobilization, diagnostic imaging, and referral decisions. For example, safely clearing patients from spinal immobilization devices is critical to prevent complications associated with prolonged use (MacCallum et al., 2020). This requires a combination of clinical expertise and access to diagnostic tools, which may not be universally available or, if available, not promptly accessible. In such cases, rigid frameworks like the currentguidelines—primarily based on evidence from HICs—may create uncertainties in the clinical workflow and lead to additional delays in patient care. For example, a previous study found that while 83 % of care providers in LMICs reported having access to MRI, the scan was obtained within 24 h in only 67 % of cases, with delays varying by income level and geographic region. Additionally, in only less than half of the cases, patients or their families did not bear the cost of diagnostic imaging. In summary, the mere availability of a diagnostic modality does not guarantee its timely and effective use. Implementing flexible management protocols may help streamline diagnostic processes and improve patient outcomes (Demetriades et al., 2022). Finally, the emergency room serves as a critical juncture for determining a patient's definitive treatment pathway, impacting overall time to care. Our protocols integrate these considerations, offering context-sensitive guidance for providers operating in diverse resource environments.

It must be mentioned that these protocols are not intended to serve as universally applicable gold-standard guidelines but rather as illustrative examples of a feasible systematic process for developing consensus-based context-sensitive management strategies. They highlight a cognitive framework that can be adapted to local contexts, considering the unique challenges and resource availability in different settings. The step-by-step documents included in the supplementary section can be efficiently modified to account for varied resource levels, serving as templates for developing local protocols. In this manner, they represent a susbtantial step toward bridging the gap between evidence-based guidelines and the realities faced in LMICs in the management of spinal trauma. By integrating experience, adaptability and evidence-informed decision-making, we hope these protocols may improve care quality, even in the most resource-constrained settings, ultimately contributing to better patient outcomes. Finally, we hope to inspire similar initiatives beyond neurotrauma and, possibly, beyond neurosurgery.

## 5 Conclusions

This study presents a set of resource-adapted protocols for the pre-hospital and emergency room management of STx and SCI. The protocols were developed through a multidisciplinary consensus-building process grounded on available evidence and aim to optimize care by tailoring step-by-step recommendations to the resources and expertise available in diverse settings. Future work should focus on evaluating the implementation and outcomes of these protocols in various settings.

## 6 Strengths and limitations

The strengths of this study lie in its innovative approach to develop resource-targeted protocols of management for STx and SCI. The protocols were created through a consensus-driven process, involving a multidisciplinary group of providers, and grounded on the best available evidence, while focusing on flexibility and adaptability.

However, while our recommendations aim to address the specific challenges faced in LMICs, they may not be universally applicable across all such settings. Healthcare systems, cultural practices, and available resources vary significantly between countries and even within regions, which may influence the feasibility and implementation of our proposed protocols.

Second, the Delphi consensus method, while a valuable tool for synthesizing expert opinions, inherently relies on the perspectives of the selected participants. This introduces a potential bias, as the experts’ experiences and regional practices may not fully capture the broader diversity of spinal trauma management across all LMICs. Despite efforts to include a diverse group of specialists, the findings should be interpreted within the context of the participants' backgrounds.

Another potential limitation is the timeframe between the literature search and the submission of this manuscript, which has allowed for some new evidence to be published in the interim. Last year, for instance, Picetti et al. published a set of 17 consensus-based recommendations on the acute management of tSCI polytrauma patients derived from a similar modified Delphi approach (Picetti et al., 2024). While several of our authors contributed to this endeavor, the practical applications of these recommendations is not universal because they do not account for differences in resource levels or provide alternatives if the preferred approach is not possible in a particular setting. Our protocols are complementary to the recommendations of Picetti et al. but additionally provide stepwise, modifiable pathways between management recommendations.

Finally, the efficacy of our protocols has yet to be determined through real-world implementation and outcome analysis. In that vein, the potential variability in adherence to the protocols remains unexplored, but is expected to be influenced by the different stakeholders and healthcare systems.

## 7 Ethics approval and consent to participate

The study was conducted in accordance with the ethical principles of the Declaration of Helsinki. Due to the nature of the study, a formal ethical approval was not required.

## 8 Competing interests

The authors declare that they have no competing interests.

## 10 Authors’ information

A group authorship (BOOTStraP-SCI study group) is present for this research paper. Authors’ information such as name, surname and affiliations are available in Supplement III.

## 11 Authors' contributions

Conception and design: NM, AD, AMR, OA.

Acquisition of data: NM, all remaining authors.

Analysis and interpretation of data: NM, all remaining authors.

Manuscript draft: NM, AMR, RMD.

Critical revision for important intellectual content: NM, AD, AMR, OA, RMD; all remaining authors.

Final approval: NM, AD, AMR, OA, RMD; all remaining authors.

## 9 Funding

This research received no specific grant from any funding agency in the public, commercial, or not-for-profit sectors. Logistical support was provided by Meditech Foundation for the live consensus meeting in Bogotà, Colombia.

## Declaration of competing interest

☒ The authors declare the following financial interests/personal relationships which may be considered as potential competing interests:

Nicolo’ Marchesini reports financial support, administrative support, and equipment, drugs, or supplies were provided by MEDITECH Foundation. The corresponding author is serving, at the time of submission, in the Editorial Board of Brain and Spine as a Reviewer Board member If there are other authors, they declare that they have no known competing financial interests or personal relationships that could have appeared to influence the work reported in this paper.
