# Supplementary material for: BOOTStrap-SCI: Beyond One option of treatment for spinal trauma and spinal cord injury: Consensus-based stratified protocols for pre-hospital care and emergency room (part I)
Source: Brain Spine. 2025 Apr 4;5:104251. doi: 10.1016/j.bas.2025.104251 (PMC12019844; doi:10.1016/j.bas.2025.104251)
Supplement: Supplementary file 1 [file mmc1.docx]

**Protocol 1: Basic Ambulance or Basic Emergency Transport – BET**

1. Verify the safety of the scene
   1. If not safe, reduce biologic and physical risks associated with the scene of injury that might affect patients and staff safety
      1. Try to establish the kinematics of trauma
   2. If safe, proceed with the next steps
      1. Try to establish the kinematics of trauma
2. Verify the presence of active severe bleedings that may put the patient’s life at risk
   1. If present, apply direct pressure on the site of bleeding with sterile bandages. Consider the use of hemostatic agents and/or torniquet if indicated and available.
   2. If not present, proceed with the next steps
3. Establish the level of consciousness using the AVPU scale (Alert – Verbal – Pain – Unresponsive). If during examination the patient shows focal neurologic deficit and/or midline spine tenderness and/or altered level of consciousness and/or intoxication and/or painful distracting injury and/or major trauma mechanism, ensure cervical spine immobilization by a hard cervical collar (except in case of penetrating neck injuries). If not available, ensure manual in-line stabilization across all the further steps
   1. If the patient responds to verbal or pain, then, confirm the patency of the airway
      1. If not patent, establish patency with the available tools
      2. If patent, proceed with the next steps
   2. If the patient does not respond, verify, and establish airways’ patency by jaw thrust maneuver and the available tools
4. Check respiratory rate and SPO2 by pulse oximetry (if available).
   1. If respiratory rate is <10 breaths per minutes or >30 breaths per minute and if SPO2 is <90%, perform 10 to 20 ventilations with a bag-valve mask (BVM or AMBU) and administer supplementary O2 to maintain an SpO2 >94%. If BVM is not available, keep the jaw thrust maneuver
   2. If respiratory rate is between 10 and 30 breaths per minute and SPO2 (if available) is >90%, proceed with the next step and check respiratory rate and SPO2 (if available) every 5 minutes
5. Perform inspection, auscultation, and palpation of the neck and thorax of the patient in order to rule out conditions that require specific interventions like a tension pneumothorax (needle decompression), open pneumothorax (Heimlich valve), massive hemothorax or cardiac hemothorax.
   1. If any of these conditions is suspected
      1. If equipment and expertise available, treat accordingly (see AET protocol)
      2. If equipment and expertise not available, keep the jaw thrust maneuver, proceed with the next steps and organize a quick transfer to the closes Emergency Department
   2. If not suspected, proceed with the next steps
6. Check the following signs of circulation:
   1. Capillary refill time:
      1. If <2 seconds (normal range) start with maintenance fluids (0.5 -1cc/Kg/h) including normal saline or ringer’s lactate.
      2. If >2 seconds (abnormal) it may be a sign of dehydration, hypothermia, hypoperfusion or hypotension, defining early use of additional crystalloid boluses (3-4cc/Kg/bolus) of normal saline or ringer’s lactate.
   2. Skin color:
      1. If normal, start with maintenance fluids (0.5 -1cc/Kg/h) including normal saline or ringer’s lactate.
      2. If pallor, sweating and cold skin might be signs of shock, defining early use of additional crystalloid boluses (3-4cc/Kg/bolus) or normal saline or ringer’s lactate.
   3. Evaluate the presence of radial pulse or measure non-invasive ABP if available.
      1. If present and systolic BP >90mmHg, start and IV access for keeping maintenance fluids (0.5 -1cc/Kg/h) including normal saline or ringer’s lactate and proceed with the next step
      2. If pulse is absent or systolic BP<90mmHg
         1. Obtain an intravenous or intraosseous access (if available) considering the level of training and the available resources,
         2. start crystalloid boluses (3-4cc/Kg/bolus) or normal saline or ringer’s lactate and re-evaluate after the bolus (considering the level of training and the available resources)
            1. If still unstable, then repeat the bolus as needed.
            2. After stabilization (return of radial pulse or systolic BP =/>90mmHg) proceed with normal saline 0.9% or Ringer Lactate (0.5 - 1mL/kg/h) as maintenance.
         3. If the patient has an associated Traumatic Brain Injury, refer to the following protocols: Recommendations of the Colombian Consensus Committee for the Management of Traumatic Brain Injury in Prehospital, Emergency Department, Surgery, and Intensive Care (Beyond One Option for Treatment of Traumatic Brain Injury: A Stratified Protocol [BOOTStraP]) and check for missing steps mainly focused in oxygenation and circulation steps.
7. Evaluate blood glucose levels (if equipment available)
   - 1. If <110mg/dL administer 15-20g of glucose
     2. If >110mg/dL proceed with the next steps
8. Check again the level of consciousness using the AVPU scale
9. Evaluate the motor and sensory function by evaluating flexion and extension of arms and hands (cervical roots) and flexion and extension of legs and feet (lumbar/sacral roots), also evaluating sensibility by dermatomes levels (C4, T4, T10, S1, L1).
   1. If motor and/or sensory deficit, repeat the above steps every 5 minutes.
   2. If neurologically normal, repeat the above steps every 15m.
10. Avoid delays and transfer the patient to the nearest and most adequate centre
    1. If the patient is hemodynamically unstable (persistent absent of radial pulse, cyanosis, or respiratory rate <10 or >30 BPM despite the treatment), transfer the patient to the nearest Emergency Room to stabilize the patient.
    2. If the patient is hemodynamically stable
       1. if there is a TBI and an abnormal motor or sensitivity exam, transfer to a facility with full capabilities for final management (CT, neurosurgery and ICU capabilities)
          1. If an adequate facility is not available, transfer the patient to the nearest Emergency Room
       2. if there is a TBI and/or an abnormal motor or sensitivity exam, transfer the patient to the nearest Emergency Room
11. Re-evaluate the patient and check motion restriction during transport
    1. If neurologic deficit, midline spine tenderness, altered level of consciousness, intoxication, or a painful distracting injury)
       1. Verify the application of a hard cervical collar (if available, If not available, continue with manual restriction until a collar is available and/or diagnostics have been performed) and a padded long backboard. The time on the backboard should be as limited as possible
    2. If no neurologic deficit, no midline spine tenderness, altered level of consciousness, intoxication, or a painful distracting injury, spinal immobilization is not necessary
12. Re-evaluate all the previous steps every 5 minutes in patients with abnormal consciousness, motor or sensitivity exams, every 15 minutes in patients with normal consciousness, motor and sensitivity exam
13. Collect relevant clinical and medical history information of the patient.

**Protocol 2: Advanced Emergency Transport - AET**

1. Verify the safety of the scene
   1. If not safe, reduce biologic and physical risks associated with the scene of injury that might affect patients and staff safety
      1. Try to establish the kinematics of trauma
   2. If safe, proceed with the next steps
      1. Try to establish the kinematics of trauma
2. Verify the presence of active severe bleedings that may put the patient’s life at risk
   1. If present, apply direct pressure on the area of bleeding with sterile bandages. Consider the use of hemostatic agents and/or torniquet if indicated and available.
      1. If bleeding is not controlled, consider the use of tranexamic acid 1g/IV diluted in 50mL of normal saline solution, and give the medication in a 10 to 20 minutes infusion.
      2. If bleeding is controlled, proceed with further steps
   2. If not present, proceed with the next steps
3. Establish the level of consciousness using the AVPU scale (Alert – Verbal – Pain – Unresponsive) or GCS. If during examination the patient shows focal neurologic deficit and/or midline spine tenderness and/or altered level of consciousness and/or intoxication and/or painful distracting injury and/or major trauma mechanism, ensure cervical spine immobilization by a hard cervical collar (except in case of penetrating neck injuries). If not available, ensure manual in-line stabilization across all the further steps
   1. If the patient responds to verbal or pain, then, confirm the patency of the airway
      1. If not patent, establish patency by cleaning and aspirating secretions, retiring foreign bodies with forceps (if available) and define indications for a nasopharyngeal airway
      2. If patent, proceed with the next step steps
   2. If the patient does not respond, verify, and establish airways’ patency by jaw thrust maneuver.
      1. If not patent, establish patency by cleaning and aspirating secretions, retiring foreign bodies with forceps (if available) and define indications for a oropharyngeal airway
      2. If patent, proceed with the next step steps
4. Check respiratory rate and SPO2 level by pulse oximetry.
   1. If respiratory rate is <10 breaths per minutes or >30 breaths per minute and if SPO2 is <90%, perform 10 to 20 ventilations with a bag-valve mask (BVM or AMBU) and administer supplementary O2 to maintain an SpO2 >94%.
      1. If SpO2 doesn’t improve by BVM ventilations, consider advanced airway management according to a rapid sequence intubation protocol.
         1. If there are difficulties to perform intubation, consider the requirement of supraglottic devices
            1. If there are difficulties in placing a supraglottic device, consider surgical airway management.
   2. If respiratory rate is between 10 and 30 breaths per minute and SPO2 (if available) is >90%, proceed with the next step and check respiratory rate and SPO2 (if available) every 5 minutes
5. Perform inspection, auscultation and palpation of the neck and thorax looking for thoracic critical injuries like tension pneumothorax, open pneumothorax, massive hemothorax or cardiac tamponade.
   1. If a tension pneumothorax is present (absent of respiratory sounds, absence of radial pulse and progressive respiratory rate >30 BPM), perform a thoracic decompression with a 14^th^ to 16^th^ gauge needle between the 4th to 5th intercostal space with middle axillary line.
      1. If an open pneumothorax is present, and the wall defect is bigger than 3cm in diameter, cover the defect with a plastic film fixed in 3 points (Heimlich valve).
         1. If symptoms improve, move to next steps.
         2. If symptoms don’t improve, check and eventually repeat the previous step
6. Check the following signs of circulation:
   1. Capillary refill time:
      1. If <2 seconds (normal range) start with maintenance fluids (0.5 -1cc/Kg/h) including normal saline or ringer’s lactate.
      2. If >2 seconds (abnormal) it may be a sign of dehydration, hypothermia, hypoperfusion or hypotension, defining early use of additional crystalloid boluses (3-4cc/Kg/bolus) of normal saline or ringer’s lactate.
   2. Skin color:
      1. If normal, start with maintenance fluids (0.5 -1cc/Kg/h) including normal saline or ringer’s lactate.
      2. If pallor, sweating and cold skin might be signs of shock, defining early use of additional crystalloid boluses (3-4cc/Kg/bolus) or normal saline or ringer’s lactate.
   3. Evaluate the presence of radial pulse and measure ABP:
      1. If present and systolic BP >90mmHg, start and IV access for keeping maintenance fluids (0.5 -1cc/Kg/h) including normal saline or ringer’s lactate and proceed with the next step
      2. If pulse is absent or systolic BP < 90mmHg
         1. Obtain and intravenous or intraosseous access
         2. Start a 250ml bolus of normal saline 0.9% and re-evaluate after the bolus.
         3. If still absence of pulse, then repeat the boluses every 5-10 minutes until recovery of the pulse.
         4. Consider the use of vasopressors if there is not improvement of pulse or systolic blood pressure >90mmHg. Use Noradrenaline amp. x 4mg / 4mL Dose: 0.05 – 0.5 mcg/kg/min or Adrenaline amp. X 1mg/mL Dose: 0.1 – 2 mcg/kg/min) and continue the transfer process.
         5. After stabilization (return of radial pulse or systolic BP =/>90mmHg) proceed with normal saline 0.9% or Ringer Lactate (0.5 - 1mL/kg/h) as maintenance..
         6. If the patient has an associated Traumatic Brain Injury, refer to the following protocols: Recommendations of the Colombian Consensus Committee for the Management of Traumatic Brain Injury in Prehospital, Emergency Department, Surgery, and Intensive Care (Beyond One Option for Treatment of Traumatic Brain Injury: A Stratified Protocol [BOOTStraP]) and check for missing steps mainly focused in oxygenation and circulation steps.
7. For pain management consider the administration of Tramadol 50mg/1mL IV/EV
8. Evaluate blood glucose levels
   - 1. If <110mg/dL administer 15-20g of glucose
     2. If >110mg/dL proceed with the next steps
     3. If the patient present seizures, administer Diazepam 10mg/IV o IM or Midazolam 10mg/IM Midazolam.
9. Check again the level of consciousness using the Glasgow Coma Scale and define if there is a neuroworsening. If there is a decrease in two or more points, consider an associated TBI and follow the Recommendations of the Colombian Consensus Committee for the Management of Traumatic Brain Injury in Prehospital, Emergency Department, Surgery, and Intensive Care (Beyond One Option for Treatment of Traumatic Brain Injury: A Stratified Protocol [BOOTStraP]) and check for missing steps mainly focused on oxygenation and circulation steps.
10. Evaluate the motor and sensory function guided by the ASIA scale and evaluating strength in the 4 extremities and the key dermatomes (C4, T4, T10, S1, L1).
    1. If ASIA A to D, repeat the above steps every 5 minutes.
    2. If ASIA E, repeat the above steps every 15m.
11. Avoid delays and transfer the patient to the closest and most adequate centre
    1. If the patient is hemodynamically unstable (persistent absent of radial pulse, cyanosis, or respiratory rate <10 or >30 BPM despite the treatment), transfer the patient to the nearest Emergency Room to stabilize the patient.
    2. If the patient is hemodynamically stable
       1. if there is a TBI and/or ASIA A-D, transfer to a facility with full capabilities for final management (CT, neurosurgery and ICU capabilities)
          1. If an adequate facility is not available, transfer the patient to the nearest Emergency Room
       2. if there is a TBI and/or ASIA E, transfer the patient to the nearest Emergency Room
12. Re-evaluate the patient and check motion restriction during transport
    1. If neurologic deficit, midline spine tenderness, altered level of consciousness, intoxication, or a painful distracting injury)
       1. Verify the application of a hard cervical collar (if available; If not available, continue with manual restriction until a collar is available and/or diagnostics have been performed)
       2. use a scoop stretcher to transfer the patient into the ambulance and for transfer from ambulance stretcher to the emergency department stretcher. Use a vacuum mattress or a long backboard with lateral head immobilizers and restriction system to the stretcher during transport. The time on the backboard should be as limited as possible
    2. If no neurologic deficit, no midline spine tenderness, altered level of consciousness, intoxication, or a painful distracting injury, spinal immobilization is not necessary
13. Re-evaluate all the previous steps every 5 minutes in patients with abnormal consciousness, motor or sensitivity exams, every 15 minutes in patients with normal consciousness, motor and sensitivity exam
14. Collect relevant clinical and medical history information of the patient.
