# Supplementary material for: BOOTStrap-SCI: Beyond One option of treatment for spinal trauma and spinal cord injury: Consensus-based stratified protocols for pre-hospital care and emergency room (part I)
Source: Brain Spine. 2025 Apr 4;5:104251. doi: 10.1016/j.bas.2025.104251 (PMC12019844; doi:10.1016/j.bas.2025.104251)
Supplement: Supplementary file 2 [file mmc2.docx]

**Protocol 1: Basic health facility (without CT)- Low Complexity**

1. Verify the presence of active severe bleedings that may put the patient’s life at risk
   1. If present, direct pressure on the site of bleeding with sterile bandages and use haemostatic agents and/or tourniquet if indicated and available
   2. If not, proceed with the next steps
2. Check airways’ patency.
   1. If not patent:
      1. Clean and aspirate secretions, retire foreign bodies with forceps (if available)
      2. Define indications for advanced airway management. If necessary:
         1. If conscious
            1. and no clinical signs of skull base fracture, use nasopharyngeal airway
            2. but with clinical signs of skull base fracture consider using nasopharyngeal airway with caution
         2. If unconscious oropharyngeal airway
   2. If patent, proceed with the next steps
3. Check respiratory rate and SPO2 level by pulse oximetry (if available).
   1. If the respiratory rate is <10 or >30 bpm, perform 10 to 20 ventilations with a bag-valve mask (BVM or AMBU) and administer supplementary O2 to maintain a SpO2 >94%.
      1. If SpO2 <90% and doesn’t improve by BVM ventilations, consider advanced airway management according to a rapid sequence intubation protocol.
         1. If immobilized, the collar should be removed and manual immobilization performed during airway intubation (by two providers, at least). One provider performs orotracheal intubation manually and the other supports the patient's head avoiding cervical mobilization.
         2. Intubation should be performed in a maximum 1 attempts with an endotracheal tube
         3. If unsuccessful, try a second attempt with a laryngeal mask, ideally with abdominal decompression (second-generation laryngeal mask, if available). If you lack experience, use a laryngeal mask.
         4. Verify successful intubation by checking 1) symmetrical expansion of the thorax 2) auscultate with the stethoscope, and 3) see that the tube passes through the vocal cords.
         5. If there are difficulties to perform advanced airway management, always consider the requirement of supraglottic devices or surgical airway management.
   2. If respiratory rate is between 10 and 30 bpm and SPO2 (if available) is >90%, proceed with the next step and check respiratory rate and SPO2 (if available) every 5 minutes
4. Perform inspection, auscultation and palpation of the neck and thorax looking for thoracic critical injuries like tension pneumothorax, open pneumothorax, massive hemothorax or cardiac tamponade.
   1. If a tension pneumothorax is present (absent of respiratory sounds, absence of radial pulse and progressive respiratory rate >30 BPM), perform a thoracic decompression with a 14th to 16th gauge needle in the 2^nd^ intercostal space in the midclavicular line or between the 4th to 5th intercostal space in the anterior axillary line.
   2. If an open pneumothorax is present, and the wall defect is bigger than 3cm in diameter, cover the defect with a plastic film fixed in 3 points (Heimlich valve).
5. Check for IV accesses. If there are no IV accesses, then establish two IV lines. Take samples for laboratory tests, including ABGs, haemoglobin level, coagulation profile, electrolytes, lactate and other required tests according to the local protocols.
6. Check again for main sources of bleeding reevaluating the thorax and evaluating the abdomen and pelvis and check again for bleeding control with direct pressure, tourniquets or pelvic bind as appropriate.
7. Check the following signs of circulation:
   1. Systolic blood pressure and mean arterial blood pressure.
      1. If hypotension (MAP <65 or SBP <90) use 3-4 cc/kg bolus of normal saline or ringer’s lactate. If available, use adrenaline or noradrenaline by micro drip boluses or infusion pumps. For patients with neurologic deficit probably due to spinal cord injury, target MAP should be between 85 and 90 mmHg
      2. If normal, proceed with the next steps
   2. Heart rate
      1. If HR<60 bpm, monitor arrhythmias or consider TBI, cardiac trauma or spinal shock.
      2. If HR >100 bpm, consider always a non-compensated hypovolemic shock. Recheck for bleeding control and/or anatomical sources of bleeding.
      3. If normal, proceed with the next steps
   3. Respiratory rate
      1. If <10 or >30 bpm, then re-evaluate the airway management or the presence of thoracic injuries without management.
      2. If normal, proceed with the next steps
   4. Check SBP and radial pulse every 5-15 minutes.
      1. If hypotension persists, continue vasoactive drugs and check the requirements of fluids to avoid excess of it (consider the presence of a combination of hypovolemic and spinal shock)
      2. If normal, proceed with the next steps
8. Check the patient for pulmonary oedema with auscultation and an early chest X-ray.
9. Place a urinary catheter. Look for a threshold of 0.5 cc/kg/h as minimum urine output. Verify before using a bladder catheter that the prostate is not floating (stands for possible urinary/bladder trauma).
10. Evaluate blood glucose levels:
    1. If <110mg/dL administer 15-20g of glucose
    2. If >110mg/dL proceed with the next steps
11. If the patient present seizures, administer Diazepam 10mg/IV o IM or Midazolam 10mg/IM Midazolam
12. Check the level of consciousness using the Glasgow Coma Scale
    1. If conscious
       1. Evaluate and annotate the motor and sensory function guided by the ASIA scale and evaluate strength in the 4 extremities and the key dermatomes (C4, T4, T10, S1, L1)
          1. If ASIA A to D
             1. Repeat the above steps every 5 minutes.
             2. Check for TBI and other major traumas

if there is no TBI or other traumas that require surgery, proceed with the application of the WHO Trauma Care Check List and perform chest and pelvic x-ray and other required imaging.

If there is TBI or other traumas, proceed with TBI management following the Recommendations of the Colombian Consensus Committee for the Management of Traumatic Brain Injury in Prehospital, Emergency Department, Surgery, and Intensive Care (Beyond One Option for Treatment of Traumatic Brain Injury: A Stratified Protocol [BOOTStraP]) and check for missing steps mainly focused on the emergency basic facility protocol

- - - - 1. Take a whole spine x-ray, if it doesn’t delay the following steps
        2. If quick access to a neurosurgeon, operative room, and ICU are available, ask immediately for neurosurgical consultation, independently from imaging findings
        3. If quick access to a neurosurgeon, operative room, and ICU are not available

If possible to organize a quick transfer

Stabilize the patient

Keep the appropriate spinal immobilization

organize the transfer as soon as possible with anesthesiologic support

If not available, consider to request support of any physician or medical figure with the competencies to follow what indicated in the ICU protocols for cardiopulmonary management

perform block movements with a minimum of 3 health providers or use scoop stretchers for moving the patient

If not possible to organize a quick transfer, maintain the above mentioned cardiopulmonary parameters until the transfer of the patient is possible

- - - 1. If ASIA E
         1. repeat the above steps every 15m.
         2. Check for TBI and other major traumas

if there is no TBI or other traumas that require surgery, proceed with the application of the WHO Trauma Care Check List and perform chest and pelvic x-ray and other required imaging.

If there is TBI or other traumas, proceed with TBI management following the Recommendations of the Colombian Consensus Committee for the Management of Traumatic Brain Injury in Prehospital, Emergency Department, Surgery, and Intensive Care (Beyond One Option for Treatment of Traumatic Brain Injury: A Stratified Protocol [BOOTStraP]) and check for missing steps mainly focused on the emergency basic facility protocol

- - - - 1. If associated neck pain or tenderness or distracting injury or not able to complete a functional range of motion of the head, take a 3-view cervical x-ray (anteroposterior, lateral and odontoid view)

If none of the above, remove cervical collar without cervical spinal imaging

- - - - 1. Screen the thoracolumbar spine according to the dynamics of trauma and clinical findings (spinal tenderness) (anteroposterior and normal lateral radiograph)
        2. According to the findings, define the need for spinal immobilization, request consultation of the general surgeons and neurosurgery and/or orthopaedics to define definitive surgical or medical management
        3. Define possibilities of observation, discharge or transfer to a higher-level facility.
  1. If unconscious:
     1. Proceed with TBI management following the Recommendations of the Colombian Consensus Committee for the Management of Traumatic Brain Injury in Prehospital, Emergency Department, Surgery, and Intensive Care (Beyond One Option for Treatment of Traumatic Brain Injury: A Stratified Protocol [BOOTStraP]) and check for missing steps mainly focused on the emergency basic facility protocol.
     2. Transfer the patient to a higher-level facility
     3. Keep the appropriate spinal immobilization until spinal imaging is obtained

1. Remove the rigid board as soon as possible due to risk of bedsores and inserting towels or other type of pads in the rigid board while transporting.
2. Collect relevant information on the medical history of the patient.
3. Avoid delays in transferring the patient to the selected facility

**Secondary Assessment in the ER** (For patients with trauma surgical indications but with delayed access to the operation room, or patients with non-surgical management indications before being transferred to the ICU or the hospitalization ward).

- 1. Repeat ASIA evaluation. If ASIA A, the patient is at higher risk of respiratory deterioration. Make emphasis on airway and ventilation management steps.
  2. Lab Tests results: If lactate >2.5meq or Base Deficit > -6, consider blood products transfusion if available (RBC, Plasma and Platelets). If the haemoglobin level < 6, perform an RBC transfusion. If INR >1.5 or PTT/PT increased over the normal lab value, then consider plasma transfusion or management with specific coagulation factors concentrates.
  3. Recheck for glucose levels. If <110mg/dL administer 15-20g of glucose. If 110mmHg proceed with the next steps.
  4. Verify that the appropriate imaging has been done according to the above-mentioned criteria
  5. Verify again the possibility of cervical clearance according to the Canadian c-spine rule
  6. Establish the referral plan to a centre with higher resources if there is no availability of neurosurgery/CT/ICU.
  7. Remove the rigid board as soon as possible due to risk of bedsores and inserting towels or other type of pads in the rigid board while transporting.

**Protocol 2: Advanced health facility/Medium–high complexity**

1. Verify the presence of active severe bleedings that may put the patient’s life at risk
   1. If present, direct pressure on the site of bleeding with sterile bandages and use haemostatic agents and/or tourniquet if indicated and available
   2. If not, proceed with the next steps
2. Check airways’ patency.
   1. If not patent:
      1. Clean and aspirate secretions, retire foreign bodies with forceps (if available)
      2. Define indications for advanced airway management. If necessary:
         1. If conscious
            1. and no clinical signs of skull base fracture, nasopharyngeal airway
            2. but with clinical signs of skull base fracture consider using nasopharyngeal airway with caution
         2. If unconscious oropharyngeal airway
   2. If patent, proceed with the next steps
3. Check respiratory rate and SPO2 level by pulse oximetry (if available).
   1. If the respiratory rate is <10 or >30 bpm, perform 10 to 20 ventilations with a bag-valve mask (BVM or AMBU) and administer supplementary O2 to maintain a SpO2 >94%.
      1. If SpO2 <90% and doesn’t improve by BVM ventilations, consider advanced airway management according to a rapid sequence intubation protocol.
         1. If immobilized, the collar should be removed and manual immobilization performed during airway intubation (by two providers, at least). One provider performs orotracheal intubation manually and the other supports the patient's head avoiding cervical mobilization.
         2. Intubation should be performed in a maximum 1 attempts with an endotracheal tube
         3. If unsuccessful, try a second attempt with a laryngeal mask, ideally with abdominal decompression (second-generation laryngeal mask, if available). If you lack experience, use a laryngeal mask.
         4. Verify successful intubation by checking 1) symmetrical expansion of the thorax 2) auscultate with the stethoscope, and 3) see that the tube passes through the vocal cords.
         5. If there are difficulties to perform advanced airway management, always consider the requirement of supraglottic devices or surgical airway management.
   2. If respiratory rate is between 10 and 30 bpm and SPO2 (if available) is >90%, proceed with the next step and check respiratory rate and SPO2 (if available) every 5 minutes
4. Perform inspection, auscultation and palpation of the neck and thorax looking for thoracic critical injuries like tension pneumothorax, open pneumothorax, massive hemothorax or cardiac tamponade.
   1. If a tension pneumothorax is present (absent of respiratory sounds, absence of radial pulse and progressive respiratory rate >30 BPM), perform a thoracic decompression with a 14th to 16th gauge needle in the 2^nd^ intercostal space in the midclavicular line or between the 4th to 5th intercostal space in the anterior axillary line.
   2. If an open pneumothorax is present, and the wall defect is bigger than 3cm in diameter, cover the defect with a plastic film fixed in 3 points (Heimlich valve).
5. Check for IV accesses. If there are no IV accesses, then establish two IV lines. Take samples for laboratory tests, including ABGs, haemoglobin level, coagulation profile, electrolytes, lactate and other required tests according to the local protocols.
6. Check again for main sources of bleeding reevaluating the thorax and evaluating the abdomen and pelvis and check again for bleeding control with direct pressure, tourniquets or pelvic bind as appropriate.
7. Check the following signs of circulation:
   1. Systolic blood pressure and mean arterial blood pressure.
      1. If hypotension (MAP <65 or SBP <90) use 3-4 cc/kg bolus of normal saline or ringer’s lactate. If available, use adrenaline or noradrenaline by micro drip boluses or infusion pumps. For patients with neurologic deficit probably due to spinal cord injury, target MAP should be between 85 and 90 mmHg
      2. If normal, proceed with the next steps
   2. Heart rate
      1. If HR<60 bpm, monitor arrhythmias or consider TBI, cardiac trauma or spinal shock.
      2. If HR >100 bpm, consider always a non-compensated hypovolemic shock. Recheck for bleeding control and/or anatomical sources of bleeding.
      3. If normal, proceed with the next steps
   3. Respiratory rate
      1. If <10 or >30 bpm, then re-evaluate the airway management or the presence of thoracic injuries without management.
      2. If normal, proceed with the next steps
   4. Check SBP and radial pulse every 5-15 minutes.
      1. If hypotension persists, continue vasoactive drugs and check the requirements of fluids to avoid excess of it (consider the presence of a combination of hypovolemic and spinal shock)
      2. If normal, proceed with the next steps
8. Check the patient for pulmonary oedema with auscultation and an early chest X-ray.
9. Place a urinary catheter. Look for a threshold of 0.5 cc/kg/h as minimum urine output. Verify before using a bladder catheter that the prostate is not floating (stands for possible urinary/bladder trauma).
10. Evaluate blood glucose levels:
    1. If <110mg/dL administer 15-20g of glucose
    2. If >110mg/dL proceed with the next steps
11. If the patient present seizures, administer Diazepam 10mg/IV o IM or Midazolam 10mg/IM Midazolam
12. Check the level of consciousness using the Glasgow Coma Scale
    1. If conscious
       1. Evaluate and annotate the motor and sensory function guided by the ASIA scale and evaluate strength in the 4 extremities and the key dermatomes (C4, T4, T10, S1, L1)
          1. If ASIA A to D
             1. Repeat the above steps every 5 minutes.
             2. Check for TBI and other major traumas

if there is no TBI or other traumas that require surgery, proceed with the application of the WHO Trauma Care Check List and perform chest and pelvic x-ray and other required imaging.

If there is TBI or other traumas, proceed with TBI management following the Recommendations of the Colombian Consensus Committee for the Management of Traumatic Brain Injury in Prehospital, Emergency Department, Surgery, and Intensive Care (Beyond One Option for Treatment of Traumatic Brain Injury: A Stratified Protocol [BOOTStraP]) and check for missing steps mainly focused on the emergency basic facility protocol

Take a whole spine CT

If thoracoabdominal angio-CT is available, taking this image should be preferred, if contrast-enhanced CT is not available, take a normal CT

- - - - 1. If quick access to a neurosurgeon, operative room, and ICU are available, ask immediately for neurosurgical consultation, independently from imaging findings
        2. If quick access to a neurosurgeon, operative room, and ICU are not available

If possible to organize a quick transfer

Stabilize the patient

Keep the appropriate spinal immobilization

organize the transfer as soon as possible with anesthesiologic support

If not available, consider to request support of any physician or medical figure with the competencies to follow what indicated in the ICU protocols for cardiopulmonary management

perform block movements with a minimum of 3 health providers or use scoop stretchers for moving the patient

If not possible to organize a quick transfer, maintain the above mentioned cardiopulmonary parameters until the transfer of the patient is possible

- - - 1. If ASIA E
         1. repeat the above steps every 15m.
         2. Check for TBI and other major traumas

if there is no TBI or other traumas that require surgery, proceed with the application of the WHO Trauma Care Check List and perform chest and pelvic x-ray and other required imaging.

If there is TBI or other traumas, proceed with TBI management following the Recommendations of the Colombian Consensus Committee for the Management of Traumatic Brain Injury in Prehospital, Emergency Department, Surgery, and Intensive Care (Beyond One Option for Treatment of Traumatic Brain Injury: A Stratified Protocol [BOOTStraP]) and check for missing steps mainly focused on the emergency basic facility protocol

- - - - 1. If associated neck pain or tenderness or distracting injury or not able to complete a functional range of motion of the head, take high-quality CT scan of the spine

If none of the above, remove cervical collar without cervical spinal imaging

- - - - 1. Screen the thoracolumbar spine according to the dynamics of trauma and clinical findings (spinal tenderness) (anteroposterior and normal lateral radiograph)
        2. According to the findings, define the need for spinal immobilization, request consultation of the general surgeons and neurosurgery and/or orthopaedics to define definitive surgical or medical management
        3. Define possibilities of observation, discharge or transfer to a higher-level facility.
  1. If unconscious:
     1. Proceed with TBI management following the Recommendations of the Colombian Consensus Committee for the Management of Traumatic Brain Injury in Prehospital, Emergency Department, Surgery, and Intensive Care (Beyond One Option for Treatment of Traumatic Brain Injury: A Stratified Protocol [BOOTStraP]) and check for missing steps mainly focused on the emergency basic facility protocol.
     2. Transfer the patient to a higher-level facility
     3. Keep the appropriate spinal immobilization until spinal imaging is obtained

1. Remove the rigid board as soon as possible due to risk of bedsores and inserting towels or other type of pads in the rigid board while transporting.
2. Collect relevant information on the medical history of the patient.
3. Avoid delays in transferring the patient to the selected facility

**Secondary Assessment in the ER** (For patients with trauma surgical indications but with delayed access to the operation room, or patients with non-surgical management indications before being transferred to the ICU or the hospitalization ward).

1. Repeat ASIA evaluation. If ASIA A, the patient is at higher risk of respiratory deterioration. Make emphasis on airway and ventilation management steps.
2. Lab Tests results: If lactate >2.5meq or Base Deficit > -6, consider blood products transfusion if available (RBC, Plasma and Platelets). If the haemoglobin level < 6, perform an RBC transfusion. If INR >1.5 or PTT/PT increased over the normal lab value, then consider plasma transfusion or management with specific coagulation factors concentrates.
3. Recheck for glucose levels. If <110mg/dL administer 15-20g of glucose. If 110mmHg proceed with the next steps.
4. Verify that the appropriate imaging has been done according to the above-mentioned criteria
5. Verify again the possibility of cervical clearance according to the Canadian c-spine rule
6. Establish the referral plan to a centre with higher resources if there is no availability of neurosurgery/ICU.
7. Remove the rigid board as soon as possible due to risk of bedsores and inserting towels or other type of pads in the rigid board while transporting.
