# Supplementary material for: BOOTStrap-SCI: Beyond One option of treatment for spinal trauma and spinal cord injury: Consensus-based stratified protocols for pre-hospital care and emergency room (part I)
Source: Brain Spine. 2025 Apr 4;5:104251. doi: 10.1016/j.bas.2025.104251 (PMC12019844; doi:10.1016/j.bas.2025.104251)
Supplement: Supplementary file 3 [file mmc3.docx]

**BOOTStraP-SCI study group**

| **Last name** | **Middle name** | **First name** | **Affiliation (s)** | **ORCiD** |
| --- | --- | --- | --- | --- |
| Adeleye | Olufemi | Amos | 1.Department of Surgery, College of Medicine, University of Ibadan, Nigeria  2.Department of Neurological Surgery, University College Hospital, UCH, Ibadan, Nigeria |  |
| Baeza | Antón | Laura | 1.Department of Neurological Surgery, Weill Cornell Medicine, Presbyterian Hospital, New York, New York, USA | 0000-0002-8062-4248 |
| Barthélemy | J | Ernest | 1.Global Neurosurgery Laboratory, Division of Neurosurgery, State University of New York (SUNY) Downstate Health Sciences University, Brooklyn, USA |  |
| Carlos | Rafael-Lanao | Peña | 1.Universidad Antonio Nariño  2.Walter E. Dandy Club - Colombia | 0000-0001-7619-9261 |
| Castillo | Luiz | José | 1.Unidad de Cuidados Intensivos Clínica Vallesalud, Cali, Colombia  2.Universidad Javeriana, Cali, Colombia |  |
| Castillo | Aragón | Nadya | 1.Servicio Nacional de Aprendizaje - SENA, Colombia | 0000-0003-1165-3923 |
| Ciro Quintero | Diego | Juan | 1.Intensive Care Service, Las Américas Clinic, Medellín, Colombia |  |
| Echeverri | Guerra | Raul | 1.Unidad de Patología Compleja de Columna, Hospital San Juan de Dios León - Castilla y León, España  2.Humankindness Organization, Venezuela |  |
| Griswold | … | Dylan | 1.NIHR Global Health Research Group on Acquired Brain and Spine Injury, Cambridge, UK  2.Department of Clinical Neurosciences, University of Cambridge Neurosurgery, Cambridge, England, UK  3.School of Medicine, Stanford University Department of Neurosurgery, Stanford, California, USA |  |
| Iaccarino |  | Corrado | 1.Department of Biomedical, Metabolic and Neural Sciences, University of Modena and Reggio Emilia, Modena, Italy.  2.Neurosurgery Unit, Neuromotor and Rehabilitation Department, Azienda USL-IRCCS of Reggio Emilia, Reggio Emilia, Italy. |  |
| Lee |  | Kiwon | 1.Department of Neurology  Director, Stroke and Neurocritical Care Program  2.Neurology Acute Care  Morristown Medical Center and Atlantic Health System |  |
| Martinez | Palacios | Karol | 1.Neurology Acute Care  Morristown Medical Center and Atlantic Health System  2.Fundación MEDITECH, Cali, Colombia | 0000-0003-1967-4761 |
| Montoya |  | Santiago | 1.Universidad Antonio Nariño  2.Universidad San Martín, Sede Sabaneta |  |
| Paiva | Silva | Wellingson | 1.Neurosurgery Division, University of São Paulo, São Paulo 14040-906, Brazil |  |
| Perez |  | Berhioska | Meditech Neuro SAS, Cali, Colombia |  |
| Pozuelos | Luiz | Julio | 1.Hospital Multimèdica, Ciudad del Guatemala, Guatemala |  |
| Reisner | … | Andrew | 1.Departments of Neurosurgery and Pediatrics, Emory University School of Medicine  Children’s Healthcare of Atlanta  Atlanta, GA 30342 USA |  |
| Robles | Vanesa | Linda | 1.Universidad El Bosque, Bogotà, Colombia |  |
| Rocabado | Aliaga | Martin | 1.Servicio de Neurocirugía, Hospital Obrero #1, La Paz, Bolivia |  |
| Sanchez | Marcela | Diana | 1.Division of Neurosurgery, Neurocentro, Pereira, Colombia  Network for Global Neurological Research | 0000-0002-8928-0613 |
| Soto | Ricardo | Alvaro | 1.Neurosurgery Service, San Antonio Departamental Hospital, Pitalito-Huila, Colombia. |  |
| Sung |  | Gene | 1.University of Southern California, Los Angeles, CA, United States |  |
| Vásquez-García |  | Sebastián | 1.Neurosciences and Intensive Care Department, Clínica del Country, Bogotá, Colombia.  2.Universidad del Rosario, Bogotá, Colombia.  3.Neurocritical Care, University of Cambridge, Cambridge, United Kingdom  4.Meditech Foundation, Cali, Colombia |  |
